# A self‐retaining mouth prop for intraoral access during periodontal surgery in rat models: A technical note

**DOI:** 10.1002/ame2.70300

**Published:** 2026-10-14

**Authors:** Saravanan Sampoornam Pape Reddy, Delfin Lovelina Francis, Vivek Kumar Thakur, Balaji Manohar, Shaili Pradhan, Shesh Rai

**Affiliations:** ^1^ Department of Periodontology Army Dental Corps Bhopal India; ^2^ Department of Public Health Dentistry, Saveetha Dental College & Hospitals Saveetha University, Saveetha Institute of Medical and Technical Sciences (SIMATS) Chennai India; ^3^ Department of Orthodontics & Dentofacial Orthopedics Military Dental Centre Devlali Maharashtra India; ^4^ Department of Periodontology, Geetanjali Dental and Research Institute Geetanjali University Udaipur India; ^5^ Department of Periodontology Kathmandu Medical College Public Limited Kathmandu Nepal; ^6^ Department of Biostatistics, Health Informatics and Data Sciences, ‘Cancer Data Science Centre’, ‘UC College of Medicine’ University of Cincinnati Cincinnati Ohio USA

**Keywords:** animal models, experimental methodology, rat oral surgery, refinement, surgical instrumentation

## Abstract

**Background:**

Rodent models are widely used in oral health research; however, maintaining stable and effective intraoral retraction in the small oral cavity is challenging. Manual retraction methods often lead to procedural instability, operator fatigue, and increased tissue trauma during rodent oral surgery.

**Methods:**

A stainless‐steel wire mouth prop incorporating helices for self‐retention and auxiliary lateral soft tissue displacement prongs was fabricated. 32 adult Wistar albino rats were randomly allocated to two groups (*n* = 16 each). In the experimental group, procedures were performed using a custom mouth prop, and the control group underwent conventional manual retraction. Outcomes included time to achieve adequate retraction, slippage events per procedure, operator fatigue scores, and complication rates. Student's *t*‐test was used for statistical analysis of retraction time, and the Mann–Whitney *U* test was used for slippage frequency, and fatigue scores. Statistical significance was determined using a Bonferroni‐adjusted threshold of *p* < 0.017.

**Results:**

Retraction time was significantly shorter in the experimental group (2.5 ± 0.6 min) than in control group (6.3 ± 1.2 min, *p* < 0.001). Slippage events per procedure were markedly lower with mouth prop (0.1 *vs*. 3.4 ± 1.1, *p* < 0.001). Operator fatigue scores were also reduced (2.4 ± 0.8 *vs*. 7.2 ± 1.4, *p* < 0.001). Complication rates were significantly lower in the experimental group (*p* = 0.024).

**Conclusion:**

The proposed custom‐made mouth prop demonstrated favorable procedural handling characteristics during rat intraoral surgery and may serve as a practical, low‐cost refinement tool for improving intraoral access and procedural stabilization in experimental oral research settings.

## Graphical abstract

A mouth prop designed to mimic the oral cavity of rats was made that provided stable, self‐retaining retraction, thereby facilitating oral surgery on rodents. Compared to conventional methods, it decreased surgical time, operator fatigue, and slippage. Its use led to higher efficiency and fewer complications and it was cost effective to produce. It thus serves as an integral device for rodent‐based oral health studies.

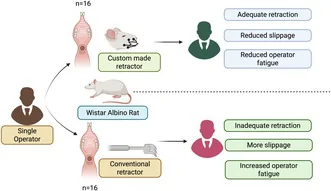

## 1 INTRODUCTION

Animal models, especially rodents such as rats, have greatly contributed to advancements in oral health sciences, including oral surgery, orthodontics, and periodontics.^1^ These models are useful for understanding human oral health conditions and evaluating pathogenesis, surgical procedures, and treatment techniques. Ensuring proper visibility and accessibility of the oral cavity during intra‐oral procedures in rodents is one of the most basic aspects of the research.^2^ Retracting the mouth successfully is complicated by the fact that the rat's oral cavity is small and closed. Retraction is critical in oral surgeries and other periodontal research procedures to provide access to the oral cavity.^3^ In addition, adequate retraction helps to keep the surgical field clean, bright, and free from unwanted mechanical obstruction, thus allowing for accurate and controlled movements. In procedures requiring the harvesting of soft/hard tissue and manipulation of bone, retraction is pivotal not only for visual access but also to minimize surgical trauma and improve overall results.^4^

The need for appropriate retraction in oral procedures is well established; however, the tools and devices currently used to achieve this in rodent models are limited and often inappropriate. For retraction, small forceps, tweezers, or basic metal clamps are the most common tools used in rodent studies.^5^ Such tools may be suitable for simple tasks, but lack the precision or extended traction required for longer procedures. Finally, they can present various complications in situations that can be encountered during a surgical procedure, such as two people being required for the procedure, in addition to making it difficult to maintain stable retraction of the oral cavity, as well as the possibility of causing trauma to the animal models due to the force exerted during the procedure. Although commercial retraction devices for rodents have been developed and patented, few exist that are not cost‐prohibitive, and they are not specifically designed to meet the unique biological architectural constraints of the rat oral cavity.^6^ Several recent studies have described commercially manufactured or custom‐engineered rodent oral retractors and mouth gags for experimental surgery and imaging applications.^7^ However, many of these systems involve specialized components, external fixation assemblies, or higher procurement costs that may limit their routine applicability in resource‐constrained laboratory settings. Furthermore, some available devices are optimized for generalized oral exposure rather than for fine periodontal or palatal microsurgical procedures that require simultaneous cheek retraction and stable self‐retention. Standardized mouth props provide a well‐defined, rigid mouth retraction, but they are difficult to adjust to different rodent sizes and not conducive to in‐house modification.^8^ In contrast to commercial devices, these mouth props were designed locally for use by researchers. Recent advances in oral surgical devices for rodents, including imaging‐compatible and simplified retractors, underscore the importance of methodological refinement in small animal models.

Therefore, there is still a practical interest in simplified, easily adaptable retraction systems that facilitate stable intraoral access during rodent oral surgical procedures, particularly in laboratories with limited access to specialized instrumentation. The present validation was intentionally restricted to a standardized palatal soft‐tissue grafting model; therefore, the findings should be interpreted in the context of procedures requiring similar microsurgical access and operative characteristics. Such a tool should be simple to fabricate using common materials, allowing it to be accessible to scientists in low‐resource laboratories. Palatal soft‐tissue procedures in rodents are technically demanding microsurgical interventions that require sustained mouth opening, precise tissue handling, unobstructed visualization, and stable access within a highly confined operative field. Accordingly, the present findings should not be extrapolated to all experimental intraoral procedures. Although the underlying principles of stable mouth opening and procedural access may also be relevant to periodontal models, these applications were not investigated in the present study and require dedicated validation before broader use can be recommended. Therefore, such procedures provide a suitable experimental platform for evaluating intraoral retraction systems and procedural stabilization devices. Therefore, this study aimed to describe and experimentally evaluate a simplified self‐retaining mouth prop intended to facilitate stable intraoral access during rodent periodontal microsurgical procedures. The device was designed as a low‐cost, easily fabricated methodological refinement tool for use in experimental oral research settings. This study aimed to enhance the efficiency and reliability of rodent‐based research in oral health sciences by providing a simple, customizable, and reliable solution for rodent oral retraction. Accordingly, we hypothesized that this simplified self‐retaining mouth prop, fabricated from readily available materials, could provide a practical methodological refinement of conventional manual retraction techniques commonly used during rodent oral surgical procedures.

## 2 METHODS

### 2.1 Study design

This study adhered to the principles of ARRIVE 2.0 guidelines.^9^ Institutional animal ethical committee clearance was obtained from Army Dental Centre (Research & Referral), Delhi, India (GU/IAEC/21/2023–24 dated 18/07/2024). This was a prospective animal study with a total sample size of 32. The sample size was determined a priori for the detection of a moderate to large effect (Cohen's *d* ≈ 0.8) with *α* = 0.05, and a power of 80%, and was estimated to be 16 animals in each group (*n* = 32). The estimate was obtained from pilot data and the literature on variability in comparable operative outcomes in rodent models, in accordance with general principles for estimating sample size in animal studies.^10^ The test and control groups were randomized at a ratio of 1:1 using block randomization of size two. Randomization was blinded to the performing surgeon using a random number table, and animals were assigned to the experimental or control group at a 1:1 ratio. Allocation was masked until the time of the operation. Operator blinding was not feasible because of the obvious physical differences between the self‐retaining mouth prop and conventional manual retraction techniques. To reduce variability, all procedures were performed using a standardized operative protocol by a single experienced operator. Study outcomes were predefined before experimentation, and objective procedural parameters, such as retraction time and slippage frequency, were prospectively recorded to minimize subjective bias wherever possible. Thirty‐two adult Wistar albino rats (*Rattus norvegicus*), comprising 16 males and 16 females, were studied. The animals were 10–12 weeks old and weighed 220–250 g at the time of the experiment. The retraction device was designed to be simple, reusable, and in‐house fabricated. All efforts were made to minimize the number of animals used, with the sample size determined through prior power calculations to ensure reliable outcomes with the fewest subjects. Measures were also taken to reduce animal discomfort. Surgical procedures were performed under isoflurane anesthesia, perioperative monitoring was conducted to identify any distress, and postoperative observation ensured no undue pain or complications. These practices were in line with ARRIVE 2.0 recommendations and institutional ethical guidelines. The study was consistent with the NIH / ARRIVE 2.0 guidelines and institutional animal ethics. Animals were kept in standard polypropylene cages with stainless‐steel grill tops, in an environment of 22 ± 2°C temperature and 55% ± 10% relative humidity, under a 12‐h light/dark cycle. Autoclaved bedding was supplied and replaced every second day. Access to a standard pelleted diet and water was available ad libitum to the rats. No attempt was made to enrich the environment at the time of surgery to minimize potential confounding factors in the surgical results. Wistar rats were chosen because of their well‐characterized anatomy and reproducibility of intraoral procedures.

### 2.2 Materials for the custom‐made rat mouth prop

Significant consideration was given to materials for the fabrication of the custom‐made rat mouth prop; it was important that the material be durable, allow for design flexibility, and withstand sterilization (autoclaving). The material used was stainless steel round wire (0.7 mm or 21 gauge). A universal plier was used to bend and shape the wire to create the basic structure of the mouth prop, including the helices, arms, and prongs. The equipment for soldering included a soldering torch, flux, and silver solder. Silver solder is favored for its bonding properties and relative convenience in metal working. To ensure that the mouth prop is adapted to the size of adult rats, precise measurement and component dimensions can be adjusted for particular experimental needs. For sterilization, the finished mouth prop was autoclaved to ensure suitability for use in rodent research settings. The performance of the rat mouth prop was validated using palatal soft tissue grafting in 32 adult Wistar albino rats (*Rattus norvegicus*).

### 2.3 Mouth prop design

The rat mouth prop was designed to be simple and adaptable. The ultimate aim was to develop a device that allows sufficient oral cavity retraction while minimizing complications related to slippage or instability during prolonged procedures.^11^ The main frame of the mouth prop was fabricated from 0.7 mm (21‐gauge) stainless steel orthodontic wire. The approximate inter‐incisal span after fabrication was 18–20 mm, with lateral cheek‐prong extensions measuring approximately 5–7 mm bilaterally. Helical retention components were constructed with approximately two turns each to provide elastic stabilization during mouth opening. Minor dimensional adjustments were made based on animal size and operative requirements. Initially, the rat mouth prop dimensions were set to a size to fit adult rats. These dimensions were chosen based on both anatomical considerations and prior investigation of rodent oral cavities. The size can be minimized by shortening the prongs' length and angle to accommodate other species or experimental conditions. The stainless‐steel wire was cut to specified lengths according to the design using universal pliers and a ruler. The wire was then shaped to the desired geometry, first by constructing the primary frame and subsequently producing helical corners. The prop uses two helices near the corners of the device. These helices are intended to maintain fixation of the oral cavity in an open position, providing a self‐retaining feature (Figure 1). The design was inspired by the mechanics of orthodontic springs widely used to provide a retracting and stabilizing force. A notch was added to the device's horizontal arms to help grip the incisors. This design tweak helps minimize slippage and locks the mouth prop in place during the procedure. The device was designed with auxiliary prongs to provide gentle lateral displacement and stabilization of the buccal soft tissues during surgery, thereby reducing soft‐tissue interference and improving access to the operative field. These prongs can be welded onto the main wire frame of the mouth prop with silver solder. The cheek prongs were designed to slowly retract the cheeks without causing extra stress or injury to the animal. Although the photograph in Figure 1 shows the cheek prongs aligned vertically at right angles to the incisal edge, in fabrication, they are placed at a slight angle to favor buccal mucosal retraction, as shown in Figure 2. This relatively stable position was suitable for exposure of the operative field. The final product was subsequently sterilized by autoclaving to ensure it was safe for use in Wistar rats.

**FIGURE 1:**
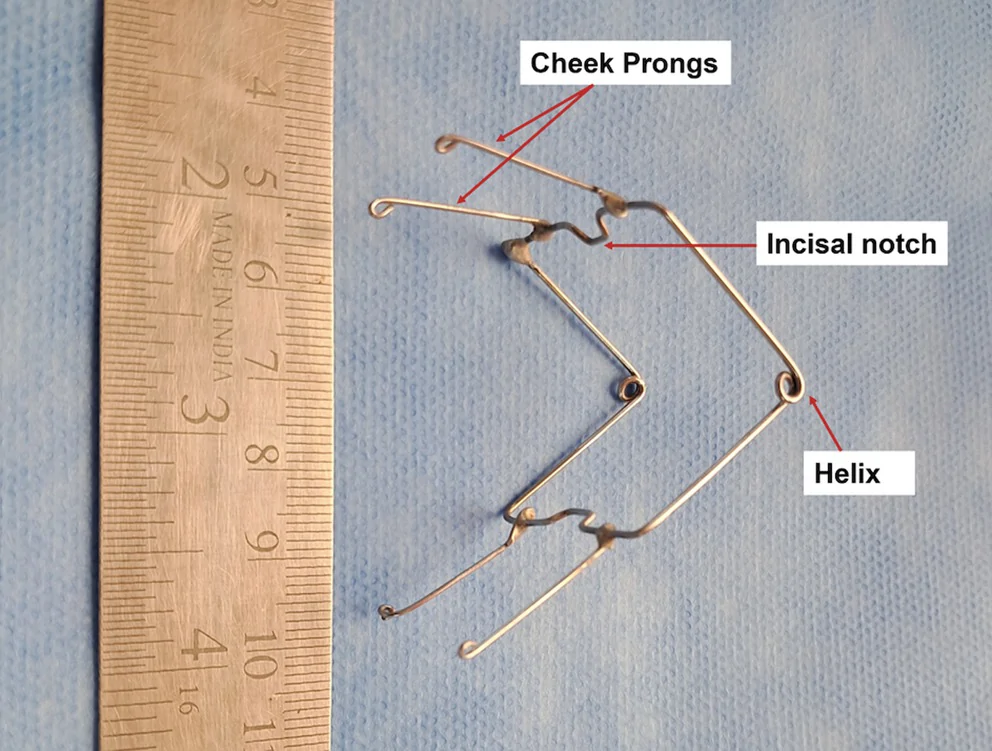
Design of the custom‐made rat mouth prop. The photograph shows the structural components of the fabricated mouth prop, including the helices for self‐retention, notched arms for incisor stabilization, and cheek‐retraction prongs. The device was constructed using stainless steel wire and silver solder. In the photograph, the cheek prongs are shown aligned vertically adjacent to the incisal notch; however, during fabrication and intraoperative use they were oriented laterally to improve buccal mucosal retraction. Scale bar = 5 mm.

**FIGURE 2:**
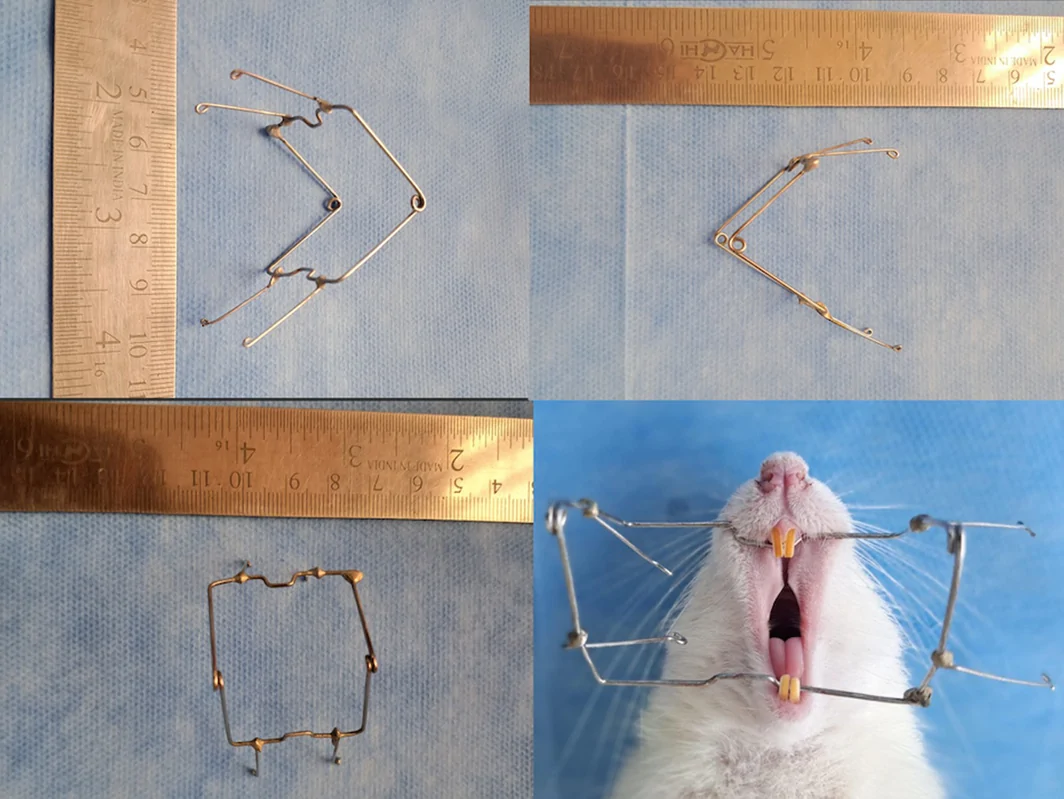
Intraoperative placement of the custom‐made rat mouth prop. Photograph demonstrating placement of the mouth prop in a Wistar rat during palatal soft tissue grafting. The device maintains a stable and wide oral opening, allowing unobstructed access to the surgical field without additional manual retraction. The auxiliary cheek prongs are oriented laterally to provide limited buccal soft tissue displacement and stabilization during palatal surgical access. Scale bar = 5 mm.

### 2.4 Validation and testing

The functional performance of the custom‐made mouth prop was evaluated during standardized palatal soft‐tissue graft harvesting procedures in Wistar albino rats. The surgical model was selected because it requires stable intraoral access, sustained mouth opening, soft tissue visualization, and precise periodontal microsurgical manipulation within the restricted rodent oral cavity. Wistar rats were anesthetized with isoflurane and positioned supine on the surgical stage. Anesthesia was initiated with 3%–4% isoflurane in oxygen in an induction chamber, and maintained with 1.5%–2% isoflurane in oxygen during the procedure. No other sedative or systemic analgesic was administered during the surgery. All animals were kept under constant monitoring during surgery to maintain anesthesia and minimize distress.^11^, ^12^ The custom‐made rat mouth prop was then gently inserted into the oral cavity to provide better stabilization of mouth opening and cheek retraction. The surgical site for soft tissue grafting was the palatal region. Briefly, the procedure involved exposure of the palatal mucosa, followed by standardized soft‐tissue grafting using microsurgical instruments. The operative procedure was used solely as a validation model to assess intraoral accessibility and device performance during periodontal surgery and was not intended to evaluate biological healing outcomes or regenerative efficacy. With the mouth prop, the operator (S.P.) had optimal access to the site, enabling him to perform the procedure without requiring further assistance. Mouth prop stability during the procedure, ease of use, and animal comfort were observed (Figure 2). Recorded outcome measurements included time to adequate retraction and stability of retraction—the number of slips or need for repositioning of the retractor over time. Operator fatigue was self‐reported and was assessed in terms of qualitative difficulty and effort during the procedure. In the control group, intraoral access was maintained using conventional manual retraction techniques, with small tissue forceps/tweezers operated by an assistant to stabilize mouth opening and soft‐tissue displacement during the procedure. No commercially manufactured self‐retaining rodent mouth gag was used in the control arm. Within the two groups, differences in these parameters were analyzed to evaluate the influence of the the rat mouth prop on the ease and efficiency of administering intra‐oral procedures. Any evidence of trauma or complications from the mouth prop used was evaluated post‐surgically.

### 2.5 Euthanasia and verification of death

At the end of the experiments, all animals were euthanized using CO_2_ inhalation displaced at approximately 20%–30% of the chamber volume per minute as per recommendations by CPCSEA^13^ and AVMA.^14^ Death was confirmed by the lack of spontaneous breathing, cardiac activity, and corneal reflex. This approach guaranteed a humane endpoint and was in accordance with the institutional procedures for animal care. All procedures involving animals and their care were conducted in accordance with the *Guide for the Care and Use of Laboratory Animals*, 8th Edition (National Academies Press, Washington, DC, 2011) and complied with institutional animal ethical committee standards.^15^

### 2.6 Statistical analysis

Statistical analyses were conducted using SPSS version 25.0 (IBM Corp., Armonk, NY, USA). Continuous variables were described as mean ± standard deviation (SD), and count or ordinal variables were reported with median and interquartile range (IQR) where relevant. Before performing inferential analyses, continuous variables were verified for normal distribution with the Shapiro–Wilk test and homogeneity of variances was determined using Levene's test. Adequate oral retraction time (a continuous variable measured in minutes) was compared between the experimental group (custom‐made mouth prop) and control group (conventional retraction), with an independent‐samples Student's *t*‐test, as data met assumptions of normality and equal variance. Retractor slippage frequency, defined as the number of retractor slippages per procedure, did not follow a normal distribution and was thus treated as count data. As such, between‐group differences in the frequency of slippage were examined using the Mann–Whitney *U* test, a non‐parametric approach suitable for small sample sizes and non‐normally distributed variables. Operator fatigue scores (a 10‐point ordinal scale, from 1 = no fatigue to 10 = maximum fatigue) were compared between groups using the Mann–Whitney *U* test because ordinal variables cannot be adequately analyzed with parametric tests. The rate of complications, as categorical data (i.e., tissue injury or difficulty with surgical access), was assessed between groups by Fisher's exact test, chosen due to the relatively small sample size. All statistical tests were two‐tailed, and a *p*‐value < 0.05 was considered statistically significant. Since three main outcomes were prespecified (retraction time, slippage frequency, operator fatigue), Bonferroni correction for multiple comparisons was performed, resulting in an adjusted significance threshold of *p* < 0.017 (0.05/3) for these variables.

## 3 RESULTS

In total, 32 adult Wistar albino rats were included (=16 per group), randomly allocated into two groups (experimental group for which intraoral procedures were performed with the custom‐made rat mouth prop); control group where conventional assistant‐dependent manual retraction using tissue forceps/tweezers was employed. All animals received palatal soft tissue grafting by a single operator. All surgical procedures were completed successfully under general anesthesia. The study outcomes focused on procedural performance metrics related to intraoral access and device handling characteristics rather than postoperative biological or regenerative endpoints.

### 3.1 Retraction time

The time to achieve adequate oral retraction was significantly shorter in the experimental group than in the controls. In the experimental group, the mean retraction time was 2.5 ± 0.6 min (95% CI: 2.18–2.82) compared to 6.3 ± 1.2 min (95% CI: 5.66–6.94) in the control group for stable exposure of the surgical field with each technique (Figure 3). An independent‐samples Student's *t*‐test comparison revealed a highly significant difference between groups (*p* < 0.001), suggesting that the custom‐made mouth prop significantly reduced the time to achieve stable intraoral retraction. A large between‐group effect size was observed for reduction in retraction time (Cohen's d ≈ 3.9), although this finding should be interpreted cautiously given the exploratory procedural nature of the study.

**FIGURE 3:**
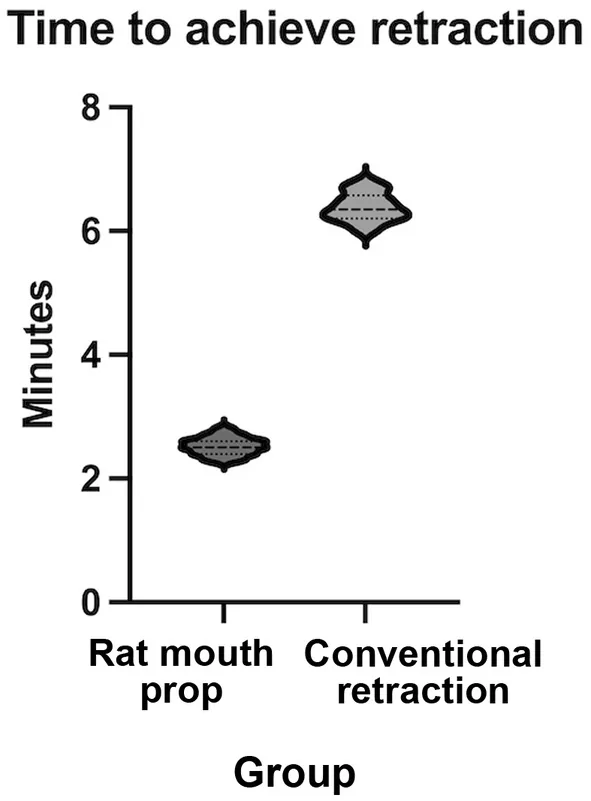
Comparison of time required to achieve adequate oral retraction between groups (*n* = 16 per group). The experimental group using the custom‐made mouth prop achieved stable retraction significantly faster (2.5 ± 0.6 min) than the control group using conventional manual retraction (6.3 ± 1.2 min). Differences were analyzed using the independent‐samples Student's *t*‐test (*p* < 0.001). Data are presented as violin plots with mean values indicated.

### 3.2 Slippage frequency

The retractor slippage rate was significantly reduced in the experimental group. Across all procedures, only two minor slippage events were recorded in the experimental group, with both being easily remedied without outside assistance (*z* = 1.4313; *p* = 0.9345). In contrast, 12 of 16 procedures in the control group experienced slippage and required additional retractor repositioning, with a mean of 3.4 ± 1.1 repositionings per procedure (Figure 4). As slippage frequency is count data, differences between groups were analyzed using the Mann–Whitney *U* test; when using the custom mouth prop, there was a statistically significant decrease in slippage events (*p* < 0.001).

**FIGURE 4:**
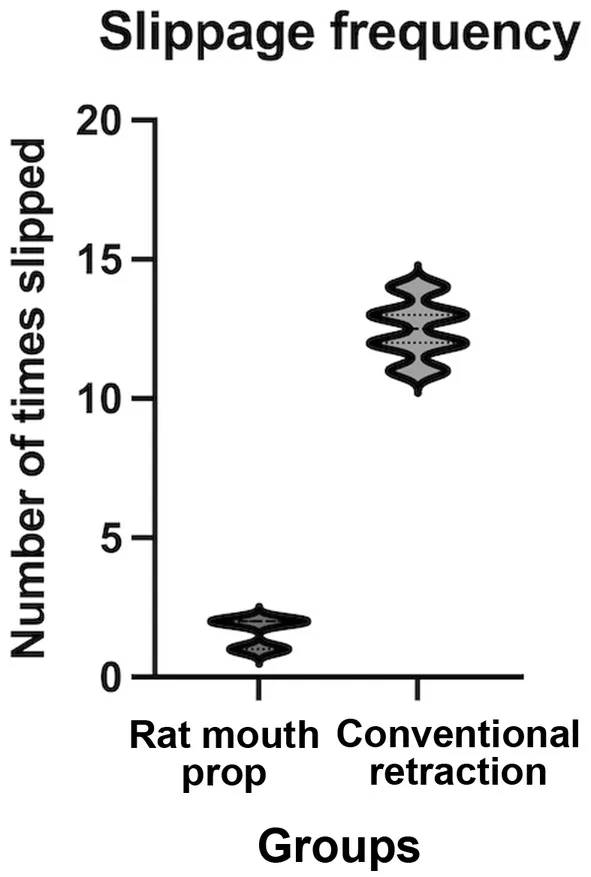
Frequency of retractor slippage events per procedure (*n* = 16 per group). Procedures performed using the custom‐made mouth prop demonstrated markedly fewer slippage events (mean 0.1 events per procedure) compared with conventional manual retraction (3.4 ± 1.1 events per procedure). Between‐group differences were analyzed using the Mann–Whitney *U* test (*p* < 0.001).

### 3.3 Operator fatigue

Operator fatigue was evaluated using a 10‐point subjective scale (1 = negligible fatigue; 10 = maximal fatigue). Procedures conducted with the custom‐made mouth prop produced significantly lower fatigue scores (2.4 ± 0.8) than conventional retraction procedures (7.2 ± 1.4) (Figure 5). Since fatigue scores are ordinal data, the comparisons were performed using the Mann–Whitney *U* test, which showed a significant decrease in operator fatigue in the experimental group (*p* < 0.001) (Figure 5).

**FIGURE 5:**
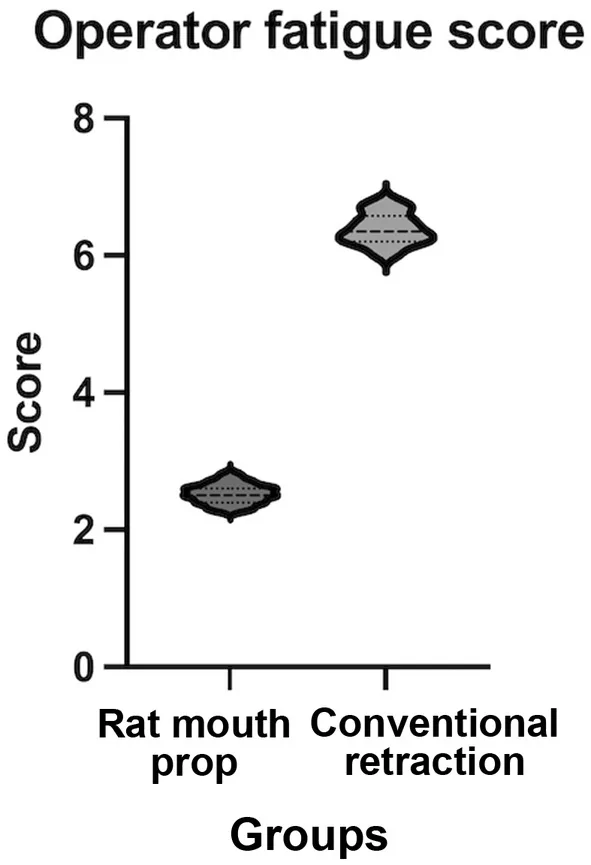
Operator fatigue scores during intraoral procedures across participants (*n* = 16/group) Operator fatigue assessed from a subjective 10‐point scale (1, minimally tired; 10, maximally tired) was significantly lower for procedures performed with the custom‐made mouth prop (2.4 ± 0.8 vs. 7.2 ± 1.4 for conventional manual retraction). Differences were assessed by Mann–Whitney *U* test (*p* < 0.001).

### 3.4 Complications

The experimental arm had fewer complications associated with retraction. The experimental group had minor mucosal abrasions in 2 cases, compared with the control group, which showed mild buccal mucosal injury in 8 cases and difficulty accessing the palatal surgical site in 5 cases. Using Fisher's exact test, there were significantly fewer complications with the custom mouth prop (*p* = 0.024).

The *p*‐values for the three prespecified primary outcomes (retraction time, slippage frequency, and operator fatigue) were statistically significant after Bonferroni correction for multiple comparisons (adjusted *α* = 0.017). Within the first few minutes after induction of anesthesia and in the early postoperative period (0–6 h), both groups of animals showed no evidence of perioperative distress, and therefore none attributable to the use of the experimental retractor.

## 4 DISCUSSION

Rodent models are widely used in oral health research, as they offer many advantages and can closely resemble human oral diseases. They are especially favorable for the investigation of periodontal disease pathogenesis and treatment.^16^ Maintaining proper and stable retraction of the oral cavity is one of the key challenges when performing intra‐oral studies in rodents, namely rats.^17^ Conventional techniques of retraction, such as employing forceps or retractors, are frequently ineffective and unsuitable for the delicate confined work of periodontal surgery. These techniques require additional manpower  to provide assistance, increase operator fatigue, and can lead to instability.^18^ The custom‐made rat mouth prop designed and fabricated in the current study offers a solution to these inherent challenges, providing self‐retaining retraction with minimal operator intervention. It also has the advantage that it significantly reduces the time taken to gain adequate retraction. Previous studies have described several rodent oral access and retraction systems, ranging from simple mouth gags to imaging‐compatible, externally stabilized retractors designed for experimental surgery and transoral procedures. However, these systems vary substantially in complexity, intended application, cost, and accessibility. The present device was therefore developed not as a replacement for these existing systems (Table 1), but as a simplified procedural refinement tool intended for rapid in‐house fabrication and routine periodontal surgical applications in small‐animal oral research settings.^19^

**TABLE 1:** Comparative characteristics of previously reported rodent intraoral retraction devices and the proposed mouth prop.

Device/Reference	Fabrication complexity	Self‐retaining	Assistant required	Relative cost^a^	Sterilizability	Primary application
Mouth gag (commercial)	Low	Yes	No	High	Yes	General oral procedures
Elastic‐assisted retractor	Moderate	Partial	Usually	Low	Variable	Oral access
Wire‐based retractors	Moderate	Yes	No	Low	Yes	Microsurgical access
Manual cheek/tongue retraction	None	No	Yes	Minimal	Not applicable	General procedures
Present custom‐made mouth prop^b^	Moderate	Yes	No	Not formally evaluated^b^	Yes	Palatal soft tissue grafting (validated); potential use in other periodontal microsurgical procedures pending validation

^a^ Cost categories are qualitative and should not be interpreted as a formal economic comparison.

^b^ The device was fabricated in a government institutional laboratory using routinely available orthodontic materials; therefore, an objective fabrication cost was not available.

The present device is also not intended as a replacement for commercially available rodent retractors or mouth gags currently used in veterinary and experimental research settings. Rather, it was designed as a simplified refinement tool for laboratories requiring rapid fabrication, procedural adaptability, and reduced dependence on specialized instrumentation. Compared with some previously described systems incorporating external stabilizers, magnetic fixators, or multi‐component assemblies, the current wire‐based design offers a lightweight self‐retaining alternative that may be particularly useful during periodontal microsurgical procedures requiring unobstructed access and single‐operator handling. The time taken for optimal retraction in the experimental group was much less than that in the control group. This time difference is due to the self‐retaining property of the custom‐made prop: once placed in situ, it does not require any additional corrections, unlike the conventional method, which requires continuous holding to keep the prop in place. In these high‐stress situations where time is of utmost importance, the improved setup and retraction times are particularly advantageous, as they shorten the procedure, which in turn reduces the likelihood of complications related to prolonged anesthesia and reduces stress on the animal model.^20^ Thus, the time saved not only allowed for improved procedural workflow but also reduced the risk of errors as well as the risk of complications during the procedure. Typically, conventional retraction techniques require repeated adjustments to ensure adequate access, which can disrupt the flow of surgery but also increases the risk of slippage or injury to the tissues.^3^ Importantly, the present study compared the proposed device against conventional manual intraoral retraction methods routinely used during rodent oral surgery rather than against commercially manufactured self‐retaining mouth gags. This comparison was intentionally selected to evaluate whether a simplified, in‐house‐fabricated device could improve procedural stability and reduce operator dependence under commonly encountered laboratory conditions. Nevertheless, direct comparative studies involving commercially available rodent retractors would further strengthen the device's external validation.

Several previously reported rodent oral retraction systems have been developed for applications including periodontal defect induction, transoral surgery, oral imaging, and intraoral prosthetic stabilization. These systems include rigid mouth gags, externally stabilized retractors, imaging‐compatible oral access devices, and multi‐component fixation assemblies. Many such devices provide excellent exposure for specialized applications; however, they may require customized fabrication infrastructure, external support systems, or specialized procurement. In contrast, the present device was designed primarily as a lightweight, self‐retaining procedural aid for routine periodontal microsurgical access, using commonly available dental materials and simplified fabrication methods.^4^, ^8^, ^17^, ^21^

The present study should be interpreted primarily as a methodological validation study rather than a therapeutic surgical investigation. The palatal grafting model was selected because it represents a technically demanding intraoral procedure that requires sustained access, stable retraction, and precise soft‐tissue manipulation within the limited rodent oral cavity. Accordingly, the principal outcomes evaluated were procedural efficiency and intraoperative handling characteristics rather than biological healing outcomes. The stability of a retraction device is an important factor  in any surgical procedure because it directly affects the surgeon's capability to perform accurate manipulations. Conventional retraction devices, such as forceps, are subject to relatively high slippage rates, requiring frequent repositioning of the retractor throughout the surgery.^19^ Our study showed slippage in only two rats out of 16 in the experimental group, which was easily corrected without any assistance. Furthermore, the frequency of slippage in the custom‐made rat mouth prop was significantly lower than that in the control group, confirming the design's accuracy and the device's stability. The self‐retaining property of the rat mouth prop contributes to its stability, as once placed in the rat's mouth, the device remains in position throughout the procedure without requiring manual holding. This allows continuous, optimal access to the surgical site while reducing the likelihood of inadvertent soft‐tissue interference and repeated tissue manipulation during the procedure; however, the present study did not directly quantify tissue injury using standardized, objective assessment methods.^22^ Using a method with a lower frequency of slippage also reduces the possibility of creating additional trauma to the animal model.^23^ The device may also contribute to improved reproducibility of rodent oral surgical models by providing standardized and stable retraction conditions across experiments. It should be emphasized that the present device was not designed to achieve maximal buccal mucosal eversion. Rather, the biomechanical purpose of the auxiliary prongs was to provide limited lateral stabilization of soft tissues during controlled mouth opening. In rodent models, excessive mouth opening may itself increase tissue tension and reduce passive cheek displacement. Therefore, the operative advantage of the present design primarily derives from stable self‐retaining mouth opening combined with modest reduction of soft tissue interference rather than extensive cheek retraction.

The other major finding of this study was that operator fatigue was reduced in the experimental group. The fatigue level was determined subjectively by the operator according to a subjective scale used by the operator to evaluate their effort and discomfort experienced during the procedure. The lower operator fatigue scores observed with the custom‐made mouth prop may be related to its self‐retaining design, which reduces the need for continuous manual stabilization during the procedure. However, because fatigue was assessed subjectively by self‐reporting, this finding should be interpreted with appropriate caution. Conventional retraction, such as with forceps or tweezers, requires sustained operator focus and handling, leading to increased physical exertion and mental exhaustion, especially during extended surgeries.^24^

Collectively, the findings demonstrate that the custom‐made mouth prop improved procedural efficiency by providing stable mouth opening, minimizing repeated intraoperative repositioning, reducing instrument slippage, and decreasing the need for continuous manual retraction. These procedural advantages resulted in shorter operating times and lower operator fatigue, thereby facilitating more consistent microsurgical access within the confined rat oral cavity. However, the present findings should be interpreted within the scope of this proof‐of‐concept validation, and further studies involving multiple operators, additional experimental models, and objective ergonomic assessments are required before broader conclusions can be drawn.^25^, ^26^ However, the present study did not directly assess postoperative pain, behavioral responses, or validated animal welfare outcomes; therefore, no conclusions regarding improved animal welfare can be drawn from these findings. Furthermore, less time spent in retraction means shorter anesthetic times, which is vital for mitigating the inevitable consequences of longer sedation.^27^

Thus, this custom‐made rat mouth prop serves as a simple, self‐retaining, and effective instrument that can be easily fabricated using commonly available materials. The adjustable design may facilitate adaptation to different experimental settings requiring stable intraoral access; however, its performance in procedures other than the palatal microsurgical model evaluated in this study remains to be established. Conventional retraction approaches, such as forceps or tweezers, are usually ineffective and tedious, especially in the small, confined rat oral cavity. This new mouth prop bypasses these constraints because of the ease of use, and the self‐retaining design that ensures constant grip. Moreover, the device's self‐retaining mechanism minimizes the need for assisting personnel, leading to higher efficiency in the research procedure and a lower risk of contamination from external contact. The prop can also be adjusted to accommodate different rodent animal sizes and experimental designs, making this a versatile tool. Additionally, the auxiliary cheek prongs provide mild bilateral soft‐tissue displacement and stabilization during surgery, helping reduce buccal tissue interference during palatal access procedures. One of the most significant benefits of this design is the ability to fabricate it using readily available materials in dental settings, such as stainless steel wire and soldering tools. This makes the mouth prop affordable for researchers in low‐budget labs. The stainless‐steel construction permits autoclave sterilization and therefore enables repeated use. It can effectively improve retraction of the rat's oral cavity during surgical operations and is easy to use. However, the long‐term stability of this device under repeated use needs to be assessed through further animal studies, compared with newer retractors. In addition, the operator should possess the technical skills to bend 21‐gauge wire into helices, arms, and prongs. Soldering equipment should also be available. This custom‐made prop was compared with conventional retraction methods that involved forceps and tweezers. It was not compared with rodent‐sized retractors that are fixed by magnets and elastomers attached to fixators.

This study addresses a relevant and practical gap in animal experiments in dentistry by proposing a low‐cost and reproducible solution for oral cavity retraction in rodent models. This study had certain limitations. The sample size was small; thus, even if sufficiently powered to detect a large effect, the results should be interpreted with caution. In addition, all surgeries were performed by a single surgeon, which may not be generalizable and does not account for inter‐observer variability. Third, we contrasted the custom‐made mouth prop with traditional manual retraction techniques and not with purpose‐built or commercial rodent retractors that may behave differently. The present study assessed operator fatigue using a subjective approach, and operator blinding was not feasible because the surgeon was necessarily aware of the retraction method being used during each procedure. Although the device was successfully sterilized by autoclaving before experimental use, the effects of repeated sterilization cycles on structural integrity, dimensional stability, retention characteristics, and mechanical performance were not evaluated. Consequently, the long‐term durability and reusability of the device remain to be established. Similarly, qualitative assessments of device handling and procedural convenience inherently involve operator judgment and should therefore be interpreted as supportive procedural observations rather than fully objective performance measures. This may have introduced performance or observational bias, particularly for subjective handling‐related outcomes. Furthermore, the fact that all surgeries were performed by a single operator may have increased the possibility of operator‐dependent effects. The present study did not include a formal economic or cost‐effectiveness evaluation. Because the device was fabricated within a government institutional laboratory using existing facilities and routinely available materials, the individual fabrication cost could not be objectively determined. In addition, tissue injury was evaluated solely by gross intraoperative observations and did not include standardized assessments of mucosal trauma, histopathological examination, or objective postoperative welfare assessments. Consequently, the present study cannot establish whether the device reduces tissue injury or improves animal welfare beyond the procedural observations reported. Although procedural standardization was maintained throughout the study, future investigations involving multiple operators, independent blinded assessors, and objective ergonomic measurements would further strengthen the reproducibility and external validity of the findings. Future studies should include operators with varying levels of microsurgical experience to assess the device's reproducibility, learning curve, and usability across users and experimental settings. Additionally, the study did not include direct benchmarking against commercially available rodent mouth gags or previously published retraction systems, which limits assessment of comparative translational performance. The large effect sizes observed in the present study should be interpreted cautiously because procedural studies involving a single experienced operator may be susceptible to operator‐dependent performance effects and observational bias. Despite the observed results and comparisons, future comparisons may also benefit from multiple operators and objective measures of fatigue, or blinded assessments wherever possible, to enhance the reliability of this outcome. Furthermore, the assumptions were not based on versions of commercially available retraction devices with distinct performance characteristics. Finally, the device's long‐term durability and repeated sterilization were not evaluated. Further evaluations with a larger sample size, including multiple operators and other retraction systems, are needed to confirm these findings.

The present findings should be interpreted within the context of a proof‐of‐concept methodological validation study. Although the device demonstrated favorable handling characteristics under the experimental conditions employed, the current work does not establish superiority over all commercially available rodent retractors or mouth gags. Rather, the proposed appliance is a simplified, alternative that uses standard laboratory materials intended for procedural stabilization and intraoral access in selected periodontal microsurgical applications. Additional validation studies incorporating direct comparisons with established commercial systems, multiple operators, objective ergonomic assessments, and enhanced visual documentation would further strengthen the evaluation of the device.

## 5 CONCLUSION

Within the limitations of this experimental study, the proposed self‐retaining rat mouth prop provided stable intraoral access with reduced need for repeated manual repositioning during palatal microsurgical procedures. The device may offer a practical tool for methodological refinement in selected rodent oral surgery applications, particularly in laboratory environments where simplified in‐house instrumentation is preferred. Further validation studies comparing the device with commercially available retraction systems are warranted.

## AUTHOR CONTRIBUTIONS

**Saravanan Sampoornam Pape Reddy:** Conceptualization; methodology; data curation; writing – original draft. **Delfin Lovelina Francis:** Investigation; validation; formal analysis; visualization; writing – review and editing. **Vivek Kumar Thakur:** Conceptualization; methodology; validation; investigation. **Balaji Manohar:** Conceptualization; methodology; validation; visualization; writing – review and editing; project administration. **Shaili Pradhan:** Validation; formal analysis; project administration; supervision; resources; writing – review and editing. **Shesh Rai:** Conceptualization; writing – review and editing; validation; methodology; project administration.

## FUNDING INFORMATION

There was no funding available for this study.

## CONFLICT OF INTEREST STATEMENT

The authors declare that there are no conflicts of interest related to this study. The authors state that this study is not funded by any funding, governmental, non‐governmental agencies or grants and there is no commercial relationship between any of the authors or any sponsorships.

## ETHICS STATEMENT

It is declared that this study obtained institutional animal ethical clearance from Institutional Animal Ethical Committee of Army Dental Centre (Research & Referral), New Delhi, India. (GU/IAEC/21/2023‐24).

## PATIENT CONSENT STATEMENT

Not applicable.

## PERMISSION TO REPRODUCE MATERIAL FROM OTHER SOURCES

Not applicable as none of the material was reproduced from any sources.

## CLINICAL TRIAL REGISTRATION

Not applicable.

## Data Availability

The data supporting the findings of this study are available from the corresponding author upon reasonable request.
